# Sex-based disparities in cardioprotective medication use in adults with diabetes

**DOI:** 10.1186/1758-5996-6-117

**Published:** 2014-11-05

**Authors:** Sonia Butalia, Adriane M Lewin, Scot H Simpson, Kaberi Dasgupta, Nadia Khan, Louise Pilote, Jeffrey A Johnson, William A Ghali, Doreen M Rabi

**Affiliations:** Department of Medicine, Faculty of Medicine, University of Calgary, Calgary, Alberta Canada; Department of Community Health Sciences, Faculty of Medicine, University of Calgary, Calgary, Alberta Canada; Faculty of Pharmacy and Pharmaceutical Sciences, University of Alberta, Edmonton, Alberta Canada; Department of Medicine, McGill University, Montreal, Quebec Canada; >Department of Epidemiology, Biostatistics and Occupational Health, McGill University, Montreal, Quebec Canada; Department of Medicine, Division of Clinical Epidemiology, McGill University, Montreal, Quebec Canada; Division of General Internal Medicine, University of British Columbia, Vancouver, British Columbia Canada; School of Public Health, University of Alberta, Edmonton, Alberta Canada; Department of Cardiac Sciences, Faculty of Medicine, University of Calgary, Calgary, Alberta Canada; Richmond Road Diagnostic and Treatment Centre, 1820 Richmond Road SW, Calgary, Alberta T2T 5C7 Canada

## Abstract

**Objective:**

The identification of sex-based disparities in the use of effective medications in high-risk populations can lead to interventions to minimize disparities in health outcomes. The objective of this study was to determine sex-specific rates of cardioprotective medication use in a large population-level administrative-health database from a universal-payer environment.

**Research design and methods:**

This observational, population-based cohort study used provincial administrative data to compare the utilization of cardioprotective medications between women and men in the first year following a diabetes diagnosis. Competing risks regression was used to calculate crude and adjusted sub-hazard ratios for time-to-first angiotensin-converting-enzyme inhibitor, angiotensin receptor blocker, or statin dispensations.

**Results:**

There were 15,120 (45.4%) women and 18,174 (54.6%) men with diabetes in the study cohort. Overall cardioprotective medication use was low for both primary and secondary prevention for both women and men. In the year following a diabetes diagnosis, women were less likely to use a statin relative to men (adjusted sub-hazard ratio [aSHR] 0.90, 95% confidence interval [CI] 0.85 to 0.96), angiotensin-converting-enzyme inhibitors (aSHR 0.90, 95% CI 0.86 to 0.94), or any cardioprotective medication (aSHR 0.93, 95% CI 0.90 to 0.97).

**Conclusions:**

Cardioprotective medication use was not optimal in women or men. We also identified a health care gap with cardioprotective medication use being lower in women with diabetes compared to men. Closing this gap has the potential to reduce the impact of cardiovascular disease in women with diabetes.

**Electronic supplementary material:**

The online version of this article (doi:10.1186/1758-5996-6-117) contains supplementary material, which is available to authorized users.

Cardiovascular disease is the leading cause of death in both women and men [1]. It is also the leading cause of morbidity and mortality for those living with diabetes. Compared to non-diabetic individuals, women and men with diabetes are two to four times as likely to develop cardiovascular disease [2]. The past two decades have witnessed the introduction of a number of therapies that are highly effective in the primary and secondary prevention of cardiovascular disease. There is robust evidence that treatment of hypertension and lipids have cardioprotective benefits independent of their respective blood pressure and lipid-lowering effects [3–7].

There is some suggestion that there is differential utilization of preventative (both primary and secondary) therapies between women and men with diabetes. Several observational studies have documented that women with diabetes have a higher risk factor burden relative to men, and others have demonstrated that women are less likely to achieve recommended targets for blood pressure, cholesterol and glucose than men [8–10]. There are small scale observational studies that do provide some evidence that women with diabetes are less likely to be treated with cardioprotective medications *and* are less likely to be treated to established therapeutic targets [9, 10]. However, it is not known if this finding of under-treatment is true at a population level which is important given the broader implications of such findings. The objective of the present study was to use a large population-level administrative-health database from a universal-payer environment to compare the use of evidence-based cardioprotective medications among women and men with diabetes.

## Research design and methods

### Data source and study population

This study was conducted using linked administrative data from Saskatchewan Health, including the subject, hospital discharge, physician visits, medical services, drug and mortality files. Approximately 90% of the Saskatchewan Health beneficiaries are eligible for prescription drug coverage. Excluded from eligibility are First Nations People and some military veterans because their prescription drugs are covered under federal programs. Persons with incomplete data or with potentially identifiable data were removed by Saskatchewan Health data administrators prior to the conduct of this work. Saskatchewan residents were eligible for inclusion if they were registered beneficiaries of Saskatchewan Health, eligible for prescription drug benefits, aged 30 years or older on the index date (date of first claim for an oral antidiabetic drug), and had continuous coverage in the provincial health plan for at least three years before the index date. Among all individuals registered with Saskatchewan health services, we identified persons with new onset diabetes defined as those who had a new oral antidiabetic prescription between January 1, 1995 and December 31, 2005. “New” prescription was defined as no antidiabetic (insulin or oral agent) therapy exposure in the year prior to the first prescription for an oral antidiabetic therapy between 1995 and 2005. Oral antidiabetic therapies are typically dispensed in 100-day supplies once a patient is in the maintenance stage, so to be eligible, subjects could have no prescriptions for antidiabetic therapies in 465 days prior to the “new” prescription. Persons with diabetes were identified by their use of antidiabetic therapy, a valid method when using comprehensive pharmacy databases [11, 12]. We excluded women using metformin alone if they had a diagnosis for polycystic ovary syndrome, and all patients with less than three years of coverage prior to the index date. All included patients were followed until December 31, 2006.

### Outcome and exposure variables

The outcome of interest was utilization of statins, angiotensin-converting-enzyme inhibitors (ACEi), and angiotensin receptor blockers (ARB). For the purposes of this study, utilization was defined as at least one dispensation for a drug in the 365 days following diabetes diagnosis. Because ACEi and ARB have similar cardioprotective effects, we also included a variable ‘ACEi or ARB’.

Our primary exposure was sex, with the analysis was stratified by age and cardiovascular disease status. Clinical practice guidelines for the care of patients with diabetes currently recommend that all patients over the age of 40 years should receive treatment with a statin, and all patients over the age of 55 years should receive treatment with and ACE or ARB [13]. A priori, we expected that patients above the age of 55 with a history of cardiovascular disease should be treated with cardioprotective medications, irrespective of sex. Patients were considered to have cardiovascular disease at baseline if there was a claim for at least one of the following in the three years prior to index date: physician office visit or hospitalization with a diagnostic code for ischemic heart disease, cardiac dysrhythmias, heart failure, cerebrovascular disease or peripheral vascular disease using the International Statistical Classification of Diseases and Related Health Problems, Ninth and Tenth Revisions; a procedure code for percutaneous coronary intervention or coronary artery bypass grafting surgery; or a prescription for a nitrate or pentoxyphylline (Additional file 1: Appendix 1).

### Statistical analysis

Baseline characteristics were compared using t-tests for continuous variables and Chi-square tests for categorical variables after assuring that assumptions of each test were met. In order to address the issue of informative censoring, competing risks regression based on the method described by Fine and Gray, with death treated as a competing event, was used to calculate crude and adjusted sub-hazard ratios for time-to-first dispensation for women relative to men [14]. Adjustment variables included age at diabetes diagnosis, cardiovascular disease status, Chronic Disease Score (quartile), renal disease, dialysis, physician office visits (quartile), hospitalizations, days in hospital, day surgeries (all) in the 3 years prior to diabetes diagnosis, year of diabetes diagnosis and pre-existing prescription for the same medication at baseline [15]. The Chronic Disease Score is a measure of co-morbidity based on pharmacy data [15]. Death and discontinuation of health services coverage (departure from the province) were considered to be competing risks when they occurred prior to a dispensation. All analyses were performed using STATA software Version 11 (StataCorp LP, College Station, Texas). The study is reported according to STROBE guidelines (Additional file 1: Appendix 2) [16].

### Ethics approval

This project received ethics approval from the Conjoint Health Ethics Review Board of the University of Calgary.

## Results

After exclusion of participants with fewer than three years of continuous coverage in the provincial health plan prior to the index date (n = 758) and those with a record of insulin dispensation in the three years prior to index date (n = 157), there were 15,120 (45.4%) women and 18, 174 (54.6%) men in the study cohort (Table 1). The mean age of women at the time of diagnosis was nominally higher than that of men (62.9 ± 15.2 versus 61.7 ± 13.2 years, p <0.001) and women had less cardiovascular disease at time of diabetes diagnosis (33.1 versus 35.8%, p < 0.0001). More men than women had a Chronic Disease Score of ≤2 (28.9% versus 20.8%, p < 0.001). Women and men differed in terms of their use of health services. Women appear to have a greater number of physician office visits, hospitalizations, and day surgeries compared to men (p < 0.001 for all comparisons; Table 1). The use of any cardioprotective medication use in the year prior to diabetes diagnosis was greater in women compared to men (38.8 versus 36.0%, p <0.0001; Table 1).

**Table 1:** **Characteristics of the study cohort**

	Men	Women	p-value
	N = 18,174	N = 15,120	
	(54.6%)	(45.4%)	
Demographic characteristics			
Mean age at DM diagnosis (SD)	61.7 (13.2)	62.9 (15.2)	<0.001
Mean age at DM diagnosis (SD) By age group			
<55	46.4 (5.9)	44.8 (6.9)	<0.001
55+	68.8 (9.0)	71.1 (9.8)	<0.001
Age group <55 years	5,766 (31.6)	4,745 (31.2)	0.457
Mean years of follow-up (SD)	5.10 (3.1)	5.14 (3.1)	0.257
Range of follow up in years	0-12.0	0-12.0	
Comorbidity			
CDS score (Quartile)			<0.001
2	5,258 (28.9)	3,143 (20.8)	
3-5	5,161 (28.4)	4,657 (30.8)	
6-8	5,004 (27.5)	4,469 (29.6)	
9+	2,751 (15.1)	2,851 (18.9)	
Cardiovascular disease status at baseline (diabetes diagnosis)	6,504 (35.8)	5,008 (33.1)	<0.0001
Renal disease in the 3 years prior to index	360 (2.0)	247 (1.6)	0.031
Access to care in the 3 years prior to index			
Total physician office visits			<0.001
0-11 office visits	5,792 (31.9)	2,857 (18.9)	
12-21 office visits	4,814 (26.5)	3,579 (23.7)	
22-35 office visits	3,976 (21.9)	4,179 (27.6)	
36 or more office visits	3,592 (19.8)	4,505 (29.8)	
Hospitalizations			<0.001
0	11,989 (66.0)	9,661 (63.9)	
1 or more	6,185 (34.0)	5,459 (36.1)	
Day surgeries			<0.001
0	13,146 (72.3)	10,587 (70.0)	
1 or more	5,028 (27.7)	4,533 (30.0)	
Cardioprotective medication use in the 1 year prior to DM diagnosis			
Statin	2,530 (13.9)	2,050 (13.6)	0.339
ACEi	4,576 (25.2)	3,831 (25.3)	0.740
ARB	1,187 (6.5)	1,401 (9.3)	<0.0001
ACEi or ARB	5,505 (30.3)	4,977 (32.9)	<0.0001
Any of the above	6,541 (36.0)	5,868 (38.8)	<0.0001

*Abbreviations:* *DM* diabetes mellitus, *SD* standard deviation, *ACEi* angiotensin-converting-enzyme inhibitors, *ARB* angiotensin receptor blockers.

Following the diagnosis of diabetes, overall cardioprotective medication use was low for women and men (Table 2). Fewer women had a statin (21.4 versus 24.0%, p < 0.0001) or ACEi (35.4 versus 39.1%, p < 0.0001) dispensation relative to men whereas women had more ARB (13.3 versus 10.3%, p < 0.0001) dispensations. Overall use of any cardioprotective medication was lower in women (53.0 versus 54.2%, p =0.03; Table 2). Upon stratification by age and cardiovascular disease status, lower proportions of women below the age of 55 had filled a prescription for a statin or ACEi and were less likely to receive any cardioprotective medication than men, irrespective of cardiovascular disease status. For those 55 years and older, once again a lower proportion of women with cardiovascular disease had filled a prescription for a statin or ACEi compared to men. In contrast, for older patients without cardiovascular disease, women were more likely to be using a statin or an ARB relative to men, and were more likely to be using any cardioprotective medication (Table 3).

**Table 2:** **Cardioprotective medication use within 1 year of diabetes diagnosis**

	Men	Women	p-value
	N = 18,174 (54.6)	N = 15,120 (45.4)	
Statin	4,368 (24.0)	3,232 (21.4)	<0.0001
ACEi	7,103 (39.1)	5,351 (35.4)	<0.0001
ARB	1875 (10.3)	2,004 (13.3)	<0.0001
ACEi or ARB	8,455 (46.5)	6,872 (45.5)	0.05
At least one of the above	9,846 (54.2)	8,011 (53.0)	0.03

*Abbreviations:* *ACEi* angiotensin-converting-enzyme inhibitors, *ARB* angiotensin receptor blockers.

**Table 3:** **Cardioprotective medication prescription within 1 year of diabetes diagnosis, by sex and cardiovascular disease status**

< 55 YEARS						
	CVD (N = 1,493 )			No CVD (n = 8,953)		
	Men	Women	p-value	Men	Women	p-value
	N = 905	N = 588		N = 4,836	N = 4,117	
Statin	366 (40.4)	180 (30.6)	0.0001	997 (20.6)	596 (14.48)	<0.0001
ACEi	431 (47.6)	225 (38.3)	0.0004	1,613 (33.4)	1,071 (26.0)	<0.0001
ARB	105 (11.6)	78(13.3)	0.338	410 (8.5)	402 (9.8)	0.035
ACEi or ARB	511 (56.5)	273 (46.4)	0.0001	1,903 (39.4)	1,382 (33.6)	<0.0001
At least one of the above	605 (66.9)	340 (57.8)	0.0004	2281 (47.2)	1608 (39.1)	<0.0001
**55 YEARS +**						
	**CVD (N = 10,019)**			**No CVD (N = 12,829 )**		
	**Men**	**Women**	**p-value**	**Men**	**Women**	**p-value**
	**N = 5,599**	**N = 4,420**		**N = 6,834**	**N = 5,995**	
Statin	1,680 (30.0)	1,142 (25.8)	<0.0001	1,325 (19.4)	1,314 (21.9)	0.0004
ACEi	2,653 (47.4)	1,931 (43.7)	0.0002	2,406 (35.2)	2,124 (35.4)	0.8
ARB	600 (10.7)	709 (16.0)	<0.0001	760 (11.1)	815 (13.6)	<0.0001
ACEi or ARB	3,084 (55.1)	2,458 (55.6)	0.6	2,957 (43.3)	2,759 (46.0)	0.002
At least one of the above	3545 (63.3)	2800 (63.4)	0.9	3415 (49.9)	3263 (54.4)	<0.0001

*Abbreviations:* *CVD* cardiovascular disease, *ACEi* angiotensin-converting-enzyme inhibitors, *ARB* angiotensin receptor blockers.

In the year following a diabetes diagnosis, women relative to men were less likely to use a statin [adjusted sub-hazard ratio (aSHR) 0.90, 95% CI 0.85 to 0.96], ACEi (aSHR 0.90, 95% CI 0.86 to 0.94), or any cardioprotective medication (aSHR 0.93, 95% CI 0.90 to 0.97; Table 4).

**Table 4:** **Sub-hazard ratio of statin, ACEi, ARB, ACEi or ARB, or any cardioprotective medication use in the 1 year following a diabetes diagnosis for women relative to men**

N = 33,294	Crude SHR (95% CI)	Adjusted* SHR (95% CI)
Statin	0.90 (0.86 – 0.95)	0.90 (0.85 – 0.96)
ACEi	0.91 (0.87 – 0.95)	0.90 (0.86 – 0.94)
ARB	1.34 (1.24 – 1.43)	1.08 (0.99 – 1.17)
ACEi or ARB	1.00 (0.97 – 1.04)	0.93 (0.89 – 0.97)
Any of the above	1.00 (0.97 – 1.04)	0.93 (0.90 – 0.97)

*Adjusted for age at diabetes diagnosis, cardiovascular disease status, Chronic Disease Score, renal disease, dialysis, physician office visits, hospitalizations, days in hospital, day surgeries (all )in the 3 years prior to diabetes diagnosis, year of diabetes diagnosis and pre-existing prescription for the same medication at baseline.

*Abbreviations:* *ACEi* angiotensin-converting-enzyme inhibitors, *ARB* angiotensin receptor blockers, *SHR* sub-hazard ratio.

## Conclusions

The results presented herein suggest that the overall use of cardiovascular medications in patients with diabetes was less than optimal. Furthermore, our analysis demonstrates that women are 10% less likely to use a statin or ACEi and are 7% less likely to use any cardioprotective medication than men after adjusting for several clinical differences that might influence the crude assessment of use. These findings lend support to the hypothesis that women with diabetes may have a greater risk, not only because of a greater risk factor burden, but also because they have less treatment of these risk factors. We acknowledge that the relative differences in use among women and men were small to moderate; however the implications of these differences on a population level could be considerable.

The finding of underuse of therapies that prevent cardiovascular disease is not novel. The recent Prospective Urban Rural Epidemiology (PURE) study similarly evaluated the use of drugs for secondary prevention of cardiovascular disease and found that use was low worldwide, with the lowest rates in low-income countries and rural areas [17]. Low cardioprotective medication use was also observed in high-income countries [17]. The socio-economic gradient in medication use documented in PURE suggests that reduced access to these therapies may be a barrier to their use. In Canada, observational studies have found similar medication underuse [18]. However, it is likely that the absolute levels of use have gone up, partly due to new clinical trial information and the aging population [18]. The relative difference in cardiovascular medication use between women and men, on the other hand, is less likely to have changed, and may be indicative of an ongoing sex/gender gap in care.

We also documented sex differences in cardioprotective medication use by age. This may not be a treatment inequity per se, as many of these differences may reflect appropriate risk stratification of individual patients and clinical judgement of treating physicians. Even though clinical practice guidelines do recommend that by age 55 all patients should be receiving statins, ACEs or ARBs, these guidelines are not sex-specific and are based on evidence from clinical trials that included largely men. Age and sex are both significant predictors of cardiovascular disease risk in all commonly used cardiovascular disease risk predication models, and women under the age of 55 will consistently have a lower predicted risk compared to men of similar age and risk factor profile. For example, using the UKPDS risk engine, a 55 year old female non-smoker with a systolic blood pressure of 140 mmHg and a total:HDL ratio of 4 will have a 10 year risk for cardiovascular disease of 10.9% [19]. A man with a similar risk factor profile will have a 10 year risk that is nearly double at 19.7%. We did not have the ability to do formal risk calculations due to the absence of clinical information such as systolic blood pressure and cholesterol levels; however, we were able to control for may clinical differences including duration of diabetes, severity of disease and co-morbid disease burden and still noted that there was a significant sex difference in cardioprotective medication use. Further, while differences in sex-related risk for cardiovascular disease may account for differences in medication use among those without established cardiovascular disease, this cannot explain the consistent finding of lower use of statins and ACEi among women with heart disease.

The decision to initiate the medications assessed in this study is admittedly more complex in women of reproductive age. These medications are all presently contraindicated in pregnant or nursing women. Should a young woman be eligible for cardioprotective therapy, either due to a high calculated risk or the presence of renal or cardiovascular disease, counselling regarding the potential teratogenicity of these medications and the need for contraception and family planning must occur. This of course should not deter the use of these therapies in younger women, but does make clinical decision making for the physician and patient more complicated. Other reasons for overall underuse of cardioprotective medications may be fear of side effects from medications, concerns of drug-drug interactions, inconvenience of laboratory surveillance, indirect costs of physician visits and prescription refills, and/or patient or physician inertia.

Our study has limitations. As it is an observational study relying on administrative data, we were limited by the data elements collected. Most importantly, we do not have clinical information such as control of glycemia, blood pressure or lipid, which may be associated with differences in medication use observed among women and men. We could at least partially address this concern by using multivariable analyses that control for severity of illness covariates defined from administrative data using validated methodologies. Ill individuals would likely be visiting their physician more frequently, may be hospitalized more and may have a higher Chronic Disease Score. We were unable to identify patients with co-morbid hypertension or nephropathy (subpopulations that would have a strong indication for the use for ACE/ARB +/− statins) in this dataset. While there are valid administrative data definitions for these conditions, the data we received had aggregated comorbidity data so we were unable to isolates specific comorbidities and were unable to calculate other commonly used co-morbidity indexes like the Charlson Comorbidity Index. We assessed medication utilization by dispensation, not actual prescriptions. Whether the difference in medication use is due to differences in physician factors (physicians underestimating women’s cardiovascular disease risk such that cardioprotective medications are not prescribed) or patient factors (patients provided a prescription that is subsequently not filled, tolerated, or adhered to) is unclear. Also, diabetes diagnosis was based on new use of antidiabetic agents. While this is an accepted method of identifying patients in pharmacy datasets, it is likely that some patients with diabetes were missed if they were managed with diet only. Further, this “new user” methodology is primarily used to define cohorts based on exposure to specific therapeutic agents and the validity of therapy use as a proxy for disease state is less established. Finally, we acknowledge that during the period of observation, the evidence for the use of statins, ACEi and ARBs was evolving and guidelines regarding their use have changed significantly over time. For much of the observation period, there were no age-specific guidelines directing use of these medications [20, 21]. More recent evidence would suggest that absolute use overall has increased [18]. The modest overall use found in our study may in part be due to appropriate low use early in the observation period when the benefits of these medications were less established or these medications were not available for use; however, we were more interested in assessing relative use in women compared to men and the relative difference in cardiovascular medication use between men and women identified herein is less likely to have changed over time. We did, nonetheless, conduct a sensitivity analysis controlling for year of entry into the cohort and our estimates regarding predictors of use and the sub-hazard ratio for use by sex or age did not change.

Our study documented significant underuse in effective cardioprotective medications in women and men with diabetes both in primary prevention and secondary prevention. Furthermore, our study documented a possible care gap in the use of cardioprotective medications in patients with diabetes, with women less likely to be treated. Significant opportunity exists to enhance cardioprotective medication use in women and men with diabetes, and efforts should be applied systemically to improve the quality of care. Future study should also be directed at assessing the clinical impact of sex differences in medication use, particularly on cardiovascular events and mortality.

## Electronic supplementary material

Additional file 1: Appendix 1: Diagnostic codes for cardiovascular diseases using the *International Statistical Classification of Diseases and Related Health Problem (ICD)*, Ninth or Tenth Revision and procedure codes for percutaneous coronary intervention or coronary artery bypass grafting surgery using the *Canadian Classification of Diagnostic, Therapeutic, and Surgical Procedures (CCP) or* Canadian Classification of Health Interventions (CCI). **Appendix 2.** STROBE Checklist. (Modified from http://www.strobe-statement.org/index.php?id=available-checklists). (DOC 98 KB)

## References

[1] Public Health Agency of Canada (2012). Cardiovascular Disease Morbidity, Mortality and Risk Factors Surveillance Information.

[2] Haffner SM (1999). Epidemiology of insulin resistance and its relation to coronary artery disease. Am J Cardiol.

[3] Hansson L, Zanchetti A, Carruthers SG, Dahlöf B, Elmfeldt D, Julius S, Ménard J, Heinz Rahn K, Wedel H, Sten Westerling for the HOT Study Group* (1998). Effects of intensive blood-pressure lowering and low-dose aspirin in patients with hypertension: principal results of the Hypertension Optimal Treatment (HOT) randomised trial. Lancet.

[4] Gæde P, Lund-Andersen H, Parving H-H, Pedersen O (2008). Effect of a Multifactorial Intervention on Mortality in Type 2 Diabetes. NEJM.

[5] Dahlöf B, Sever PS, Poulter NR, Wedel H, Beevers DG, Caulfield M, Collins R, Kjeldsen SE, Kristinsson A, McInnes GT, Mehlsen J, Nieminen M, O'Brien E, Ostergren J, ASCOT Investigators (2005). Prevention of cardiovascular events with an antihypertensive regimen of amlodipine adding perindopril as required versus atenolol adding bendroflumethiazide as required, in the Anglo-Scandinavian Cardiac Outcomes Trial-Blood Pressure Lowering Arm (ASCOT-BPLA): a multicentre randomised controlled trial. Lancet.

[6] UK Prospective Diabetes Study Group (1998). Tight blood pressure control and risk of macrovascular and microvascular complications in type 2 diabetes: UKPDS 38. BMJ.

[7] The Heart Outcomes Prevention Evaluation Study Investigators (2000). Effects of an Angiotensin-Converting–Enzyme Inhibitor, Ramipril, on Cardiovascular Events in High-Risk Patients. N Engl J Med.

[8] Gouni-Berthold I, Berthold HK, Mantzoros CS, Böhm M, Krone W (2008). Sex Disparities in the Treatment and Control of Cardiovascular Risk Factors in Type 2 Diabetes. Diabetes Care.

[9] Wexler DJ, Grant RW, Meigs JB, Nathan DM, Cagliero E (2005). Sex Disparities in Treatment of Cardiac Risk Factors in Patients With Type 2 Diabetes. Diabetes Care.

[10] Nau D, Mallya U (2005). Sex Disparity in the Management of Dyslipidemia Among Patients With Type 2 Diabetes Mellitus in a Managed Care Organization. Am J Manag Care.

[11] Ray WA (2003). Evaluating Medication Effects Outside of Clinical Trials: New-User Designs. Am J Epidemiol.

[12] Schneewiess S, Suissa S (2013). Textbook of Pharmacoepidemiology, Second Edition. Advanced Approaches to Controlling Confounding in Pharmacoepidemiology Studies.

[13] Canadian Diabetes Association Clinical Practice Guidelines Expert Committee (2008). Canadian Diabetes Association 2008 clinical practice guidelines for the prevention and management of diabetes in Canada. Can J Diabetes.

[14] Fine J, Gray R (1999). A proportional hazards model for the subdistribution of a competing risk. J Am Stat Assoc.

[15] Von Korff M, Wagner EH, Saunders K (1992). A chronic disease score from automated pharmacy data. J Clin Epidemiol.

[16] Elm E, Altman DG, Egger M, Pocock SJ, Gøtzsche PC, Vandenbroucke JP (2007). Strengthening the reporting of observational studies in epidemiology (STROBE) statement: guidelines for reporting observational studies. BMJ.

[17] Yusuf S, Islam S, Chow CK, Rangarajan S, Dagenais G, Diaz R, Gupta R, Kelishadi R, Iqbal R, Avezum A, Kruger A, Kutty R, Lanas F, Lisheng L, Wei L, Lopez-Jaramillo P, Oguz A, Rahman O, Swidan H, Yusoff K, Zatonski W, Rosengren A, Teo KK, Prospective Urban Rural Epidemiology (PURE) Study Investigators (2011). Use of secondary prevention drugs for cardiovascular disease in the community in high-income, middle-income, and low-income countries (the PURE Study): a prospective epidemiological survey. Lancet.

[18] Jackevicius CATK, Filate WA, Brien SE, Tu JV (2003). Canadian Cardiovascular Outcomes Research Team. Trends in cardiovascular drug utilization and drug expenditures in Canada between 1996 and 2001. Can J Cardiol.

[19] Diabetes Trials Unit (2012). UKPDS Risk Engine.

[20] American Diabetes Association (2003). Management of dyslipidemia in adults with diabetes. Diabetes Care.

[21] Arauz-Pacheco C, Parrott MA, Raskin P, American Diabetes Association (2003). Treatment of hypertension in adults with diabetes. Diabetes Care.

